# The Role of Magnesium in the Pathogenesis and Treatment of Glaucoma

**DOI:** 10.1155/2014/745439

**Published:** 2014-10-13

**Authors:** Feyzahan Ekici, Şafak Korkmaz, Emine Esra Karaca, Sabahattin Sül, Hasan Ali Tufan, Bahri Aydın, Ergin Dileköz

**Affiliations:** ^1^Department of Ophthalmology, Recep Tayyip Erdoğan University Medical School, 53020 Rize, Turkey; ^2^Department of Ophthalmology, Duzce State Hospital, 81100 Duzce, Turkey; ^3^Department of Ophthalmology, Sorgun State Hospital, 66700 Yozgat, Turkey; ^4^Department of Ophthalmology, Yatağan State Hospital, 48500 Muğla, Turkey; ^5^Department of Ophthalmology, Onsekizmart University Medical School, 17020 Canakkale, Turkey; ^6^Department of Ophthalmology, Gazi University Medical School, 06560 Ankara, Turkey; ^7^Department of Medical Pharmacology, Gazi University Medical School, 06560 Ankara, Turkey

## Abstract

Glaucoma is characterized by chronic optic neuropathy resulting in progressive vision loss. Not only is glaucoma considered as a condition of elevated intraocular pressure (IOP), but also other risk factors may play a role in the pathogenesis of glaucomatous optic nerve damage. Vascular dysregulation in ocular blood flow and oxidative stress are currently suggested as important risk factors for glaucomatous retinal ganglion cell loss. New treatment modalities that improve ocular blood flow and reduce oxidative stress have been investigated in many studies. Magnesium (Mg) is thought to be one of the molecules that has a treatment potential in glaucoma. Mg has been shown to improve blood flow by modifying endothelial function via endothelin-1 (ET-1) and endothelial nitric oxide (NO) pathways. Mg also exhibits neuroprotective role by blocking N-methyl-D-aspartate (NMDA) receptor-related calcium influx and by inhibiting the release of glutamate, and hence protects the cell against oxidative stress and apoptosis. Both improvement in ocular blood flow and prevention of ganglion cell loss would make magnesium a good candidate for glaucoma management. Further studies on the effect of Mg may open a new therapeutic era in glaucoma.

## 1. Introduction

Glaucoma is characterized by chronic optic neuropathy resulting in progressive vision loss [1]. IOP has been considered as the main risk factor for glaucoma; thus, medical or surgical IOP lowering therapeutic modalities play an essential role in the management of glaucoma [2]. However, IOP, as the only risk factor for glaucoma, has been considered inadequate to reveal all the potential underlying mechanisms. The majority of people with increased IOP do not develop glaucoma; on the other hand, about half of the patients with glaucomatous optic neuropathy (GON) have IOP in the normal range [3]. Furthermore, the reduction of IOP improves the prognosis of GON but does not prevent progression in all patients [2]. IOP lowering treatment is excellent in patients with angle-closure glaucoma [2], good in primary open angle glaucoma (POAG) [4], and modest in normal tension glaucoma (NTG) [5]. Therefore, other risk factors have gained importance and may have a role in the prevention and treatment of glaucoma.

Disturbed ocular blood flow and oxidative stress are the suggested concomitant risk factors that may contribute to GON [2, 3]. Blood flow reduction was claimed to be more prominent in patients with NTG than with high tension glaucoma and may be more pronounced in progressive types of glaucoma in comparison to stable forms [6, 7]. Therefore, many medications have been studied for their potential of clinical use depending on their efficiency in the regulation of ocular blood flow and the reduction of oxidative stress. Carbonic anhydrase inhibitors have been reported to improve ocular blood flow and visual field parameters in patients with glaucoma [8]. A similar improvement in ocular blood flow and visual field was also observed in patients with vascular dysregulation after being treated with calcium channel blockers [9–12]. Another pharmacological agent, dipyridamole, a platelet inhibitor, has been shown to improve ocular blood flow in a group of patients with impaired ocular blood flow including glaucoma, anterior ischemic optic neuropathy, vasospastic syndrome, or central retinal vein occlusion [13]. In terms of decreasing oxidative stress, aminoguanidine, an oral insulin stimulant for type 2 diabetes mellitus and a specific inhibitor of inducible nitric oxide synthase (NOS-2), was experimentally shown to prevent the development of GON [14]. Ginkgo biloba extract, an antioxidant polyphenolic flavonoid, has been reported to improve visual field parameters in a double-blinded placebo-controlled study [15]. Ginkgo biloba extract was shown to protect the mitochondria from oxidative stress and thereby might rescue the retinal ganglion cells [16]. Mg may add a therapeutic value in the field of glaucoma via similar mechanisms such as improvement in ocular blood flow, reduction of oxidative stress, and neuroprotection.

Mg is involved in many metabolic processes such as maintenance of normal cell membrane function, energy metabolism, and synthesis of nucleic acids [17]. Additionally, Mg acts as a natural physiologic calcium channel blocker and is part of many enzymes which play important roles in carbohydrate, protein, and fat metabolism [17, 18]. Particularly, Mg has been shown to improve the ocular blood flow in patients with glaucoma and may protect the retinal ganglion cell against oxidative stress and apoptosis [2, 3, 19]. Thereby, Mg, exhibiting beneficial effects through both neuronal and vascular mechanisms, may serve as an attractive therapeutic agent in glaucoma.

## 2. Physiological and Pharmacological Effects of Magnesium

Mg is the second most abundant intracellular cation and has been recognized as a cofactor in more than 300 enzymatic reactions in the body. Approximately 50% of Mg is present in bones, 50% in tissues and organs, and 1% in the blood stream [20]. Some of the processes in which Mg is a cofactor included, but are not limited to, protein synthesis, cellular energy production and storage, reproduction, DNA and RNA synthesis, and mitochondrial membrane stabilization [21]. Magnesium also plays a critical role in maintaining normal nerve and muscle function, cardiac excitability (normal heart rhythm), neuromuscular conduction, muscular contraction, vasomotor tone, normal blood pressure, bone integrity, and glucose and insulin metabolism [21–23]. Moreover, physiological extracellular Mg was shown to potentiate adenosine-mediated inhibition of glutamate release, restore blood-brain barrier integrity, and noncompetitively antagonize NMDA receptor activation via blockage of voltage-dependent calcium channels in the nervous system [24]. In this regard, Mg deficiency has been associated with a number of neurological diseases, including migraine, Alzheimer's diseases, and cerebrovascular diseases, and Mg therapy has been shown to have neuroprotective effects after spinal cord injury, lowering the risk of thromboembolic events due to its antithrombotic activity [24].

Normal serum Mg level ranges between 1.7 and 2.2 mg/dL, and approximately 20% of this cation is bound to albumin in the intravascular compartment. Under basal conditions, small intestine absorbs 30–50% of Mg intake and although 80% of serum Mg is filtered at the glomerulus, only 3% is finally excreted in the urine [25]. Mg therapy is associated with some minor side effects such as flushing, nausea and vomiting, muscle weakness, dizziness, and somnolence. Serious side effects which included loss of patellar reflex and respiratory depression are very rare and occur generally after parenteral application at higher doses (>13 mEq/L). Calcium gluconate is administered to counteract the effect of Mg in case of levels above the therapeutic range [26].

## 3. Magnesium Deficiency and the Impact on Ocular Tissues

In experimental animal studies, it has been shown that Mg has a vital role in the development and normal functioning of the eye. Mg deficiency has been associated with multifocal necrosis in the retinal pigment epithelium of rats [27] and hypomagnesemia was also found to be correlated with pigmentary retinal degenerations like Kearns-Sayre syndrome and retinitis pigmentosa [28, 29]. Additionally, in experimental rats Mg deprivation during developmental phase may cause multifocal necrosis and myelination disorders in the optic nerve [30]. Furthermore, Mg might be necessary for maintenance of healthy ocular surface in the prevention of infections and dryness and in inflammatory conditions of conjunctiva and cornea local application of Mg sulphate has shown some benefits [31]. Additionally, in Mg deficient rats decreased microvilli in corneal epithelial cells and apoptosis-like nuclear changes in corneal epithelial and endothelial cells were observed [32]. On the other hand, patients with keratoconus have also been shown to have low serum Mg levels [33]. Furthermore, Mg taurate has been reported to reduce the progression of cataracts [34]. Regarding the relationship of Mg with retinal disorders, patients with diabetic retinopathy were reported to have low serum Mg levels and those with the severest degree of retinopathy had more prominent hypomagnesemia [35]. Mg deficiency may have a causative relation with several disorders of the eye and may point out a potential therapeutic value.

## 4. Ocular Blood Flow, Vascular Dysregulation, and the Role of Magnesium in Glaucoma

Vascular dysregulation, an imbalance between vasoconstriction and vasodilatation at microcirculatory level and general dysfunction of endothelium and autonomic nervous system, was believed to be a contributing factor for GON [36]. Reduction in ocular blood flow was reported in various ocular tissues including the retina, optic nerve, iris, and choroid in patients with glaucoma [37–39]. Additionally, ischemia may damage the outflow system, in particular the trabecular meshwork, and thereby may increase IOP [40]. Vascular dysregulation syndromes were divided into two groups as primary and secondary vascular dysregulation syndromes. Primary vascular dysregulation is a tendency to respond differently to stimuli such as coldness or emotional stress. On the other hand, secondary vascular dysregulation syndrome is a dysfunction of anatomically normal vessels which are secondarily induced by an underlying disease [41]. Primary vascular dysregulation, associated with low blood pressure, increased venous pressure, and disturbed autoregulation, was postulated as a major cause of vascular dysfunction in the pathogenesis of GON. In individuals with primary vascular dysregulation, ocular blood flow tends to be unstable, and IOP fluctuates above and blood pressure fluctuates below the normal capacity of autoregulation. Patients with glaucoma in general may have a diminished blood flow but it is rather instability in blood flow that leads to glaucomatous damage [41, 42].

When autoregulation is disturbed, even normal IOP or normal blood flow fluctuation leads to ocular blood flow fluctuation outside the normal limits, contributing to optic nerve damage [41]. The balance of the autonomic nervous system was found shifted towards sympathetic activity in patients with NTG [43] and their choroidal vessels were observed to constrict more than normal ones [44]. Insulin resistance, a known factor of sympathetic overactivity [45], has also been reported in association with the pathogenesis of glaucoma. Patients with glaucoma or central retinal vein occlusion were demonstrated to be hyperinsulinemic compared to the control group [46]. In addition, insulin resistance has been shown to correlate positively with increasing IOP [47]. Furthermore, with proper treatment of insulin resistance-associated risk factors such as dyslipidemia, systemic hypertension, and obesity, significant IOP reduction was observed [4]. Mg has been believed to play an important role in insulin homeostasis. Dietary Mg intake and serum Mg levels were found to be inversely correlated with fasting serum insulin levels [23]. Additionally, plasma Mg concentration is known to be lower in diabetic than in nondiabetic patients [48], and hypomagnesemia has been found to be associated with diabetic retinopathy [35]. Furthermore, metabolic syndrome, a cluster of insulin resistance and glucose intolerance, visceral obesity, dyslipidemia, and hypertension, has been found to be associated with glaucoma, even though several studies have revealed no association between glaucoma and diabetes mellitus [49–51]. In subjects with metabolic syndrome and type 2 diabetes mellitus, inflammatory mediators and cytokines in adipose tissue induced endothelial dysfunction and oxidative stress which has been believed to be one of the contributing mechanisms of glaucoma [52, 53]. Therefore, glaucoma may have a relation with metabolic syndrome, insulin resistance, or diabetes-related inflammation. Consequently, endothelial dysfunction, insulin resistance, and chronic inflammation, as a result of metabolic syndrome, could be the revealing mechanisms of disturbed ocular blood flow. Mg deficiency was found to be associated with metabolic syndrome and related systemic inflammation and endothelial dysfunction [19]; thereby Mg supplementation, as an add-on therapy, may have a therapeutic value in glaucoma and may protect the ocular tissues by regulating the ocular blood flow and reducing oxidative stress. This could be supported by the finding that Mg enhances ocular vasodilation and optimizes the ocular blood flow by reduction of cytokine and free radical production and prevention of intracellular calcium entry which minimizes ganglion cell injury and neuronal loss [54].

Ocular perfusion instability occurs especially when IOP or blood pressure fluctuates and causes a repetitive reperfusion injury resulting in chronic oxidative stress, especially in mitochondrial level [2]. It was suggested that oxidative stress was involved in the pathogenesis of glaucoma [55] and unstable ocular blood flow may lead to the production of free oxygen radicals and oxidative damage in the retinal ganglion cells [2]. Oxidative stress is known to upregulate the synthesis of neuronal and inducible NOS, leading to overproduction of NO, which acts as a neurodestructive agent by the production of peroxynitrites. During reperfusion injury, a high concentration of superoxide radicals and NO results in the formation of highly damaging peroxynitrites, [56] which may lead to retinal ganglion cell loss and GON [2]. In addition, increased NO damages the membrane permeability by the nitrosylation of gap junctional proteins [57]. Besides triggering inducible and neuronal NOS, oxidative stress is also known to increase the levels of endothelin-1 (ET-1) which is a vasoconstrictor acting against vasorelaxants in the autoregulation of optic nerve head blood flow. On the other hand, higher plasma ET-1 levels were reported in patients with glaucoma despite a normalized IOP [58]. Additionally, aqueous humor ET-1 levels were found elevated in high tension glaucoma [59]. Increased ET-1 not only reduces optic nerve blood flow and interferes with axoplasmic transport but also activates astrocytes [60]. Once activated, astrocytes upregulate the production of free radicals, NOS and ET-1, thereby creating a microenvironment leading to axonal damage [3]. Mg also indirectly inhibits the activation of astrocytes and prevents neuronal loss via the inhibition of endothelin pathway [61]. Thereby, with reduction of oxidative stress and vasorelaxation, the role of Mg should further be investigated in the prevention and treatment of glaucoma and glaucoma-related neuronal loss.

Vascular tone is regulated locally by endothelium derived vasoactive agents such as ET-1 and endothelial NO. ET-1 causes the contraction of the retinal and optic nerve head vessels which is dependent on extracellular calcium influx through voltage-gated calcium channels, resulting in decreased ocular blood flow and ischemia [62]. ET-1 may be effectively inhibited by calcium channel blockade; therefore, calcium channel blockers were investigated for the management of glaucoma. In this sense, some calcium channel blockers have shown a positive effect on ocular blood flow and visual field in NTG [9, 10, 12, 63]. But systemic side effects such as hypotension and bradycardia restricted the use of calcium channel blockers for the treatment of glaucoma [64]. However, Mg acts as a natural physiologic calcium channel antagonist with minimal cardiovascular side effects and tends to increase blood flow and decreases vascular resistance in various vascular beds [65, 66]. Elevated levels of Mg inhibit ET-1 induced contraction in porcine ciliary arteries and may regulate the perfusion abnormality at microcirculatory level [67]. Therefore, Mg supplementation may have a therapeutic effect by decreasing the ocular vascular tonus via inhibition of the ET-1 induced contraction. Besides acting as a physiological calcium antagonist, Mg also has a direct vasorelaxant activity [68]. Furthermore, Mg therapy has been shown to improve endothelial function [65, 69] and induce endothelium-dependent vasodilation mediated by endothelial NO [70]. Unlike inducible NOS (NOS-2), endothelial NOS (NOS-3), a major regulator of vascular hemodynamics, may act as a neuroprotective agent [71]. Endothelial NO, derived from endothelial nitric oxide synthase (NOS-3), may protect ganglion cells by causing vasodilation and increasing ocular blood flow in patients with glaucoma [71].

## 5. Oxidative Stress and Role of Magnesium in Neuroprotection

Vascular dysregulation and repeated reperfusion injury does not only activate astrocytes and endothelin pathway but also lead to glutamate retention in retinal tissue as a result of increased oxidative stress [72]. It is known that glutamate is the major excitatory neurotransmitter in central nervous system and in retina [72], and chronic low dose glutamate exposure may damage retinal ganglion cells [73]. Glutamate excitotoxicity and oxidative stress have been suggested as one of the important pathophysiological mechanisms in glaucomatous neurodegeneration [74, 75]. The glutamate-induced excitotoxicity in neuronal tissues is mediated through NMDA receptors which are coupled with voltage-gated calcium channels [76]. At normal resting membrane potential, the pores of these channels are blocked with Mg ions. In Mg deficiency, NMDA receptors allow excess influx of calcium ions [19]. Additionally, overstimulation of NMDA receptors with excessive glutamate leads to excess calcium influx via NMDA receptor coupled calcium channels and results in the accumulation of excessive intraneuronal calcium [77]. Calcium overload causes cellular swelling and cell death [19]. Furthermore, excess calcium influx also causes oxidative stress with the generation of neurotoxic free radicals and NO [77]. Calcium influx into retinal ganglion cells seems to have a major role in producing permanent cellular damage and calcium channel antagonists have been shown to decrease NMDA-related neurotoxicity in retinal ganglion cells [78].

As we discussed, Mg, as a physiological calcium channel blocker, may be a good candidate in the protection of retinal ganglion cells. Mg ions regulate the conductance of calcium channels and limit neuronal calcium influx. Additionally, Mg inhibits the release of glutamate and thereby may participate in the protection of retinal ganglion neuronal cells against oxidative stress and apoptosis [78]. Furthermore, Mg is required for glutathione biosynthesis and its depletion has been associated with decreased antioxidant activity [79]. Mg also demonstrates antioxidant activity in retinal tissue by influencing superoxide dismutase [80]. Other than these pathogenetic interactions, Mg is one of the most important cofactors of several enzymatic reactions, involving Na^+^/K^+^ ATPase, which is responsible for the active transport of Na^+^ out of the cell membrane in exchange with K^+^ [81]. Mg deficiency leads to functional loss of Na^+^/K^+^ ATPase and causes intracellular Na^+^ accumulation and release of mitochondrial Ca^++^ ions which results in increased cytoplasmic Na^+^ and Ca^++^ [82]. Increased intracellular Ca^++^ and Na^+^ further cause cellular swelling leading to retinal ganglion cell apoptosis. In summary, Mg may protect retinal ganglion cells from oxidative injury by combined effects on voltage-dependent calcium channels, glutathione synthesis, lipid peroxidation, and maintaining the regulation of many metabolic enzymatic reactions.

## 6. Clinical Reflections

Mg supplementation-induced favourable vascular effects have been demonstrated in diabetic patients and patients with coronary artery disease [65, 69]. Therefore, Mg therapy was believed to have a therapeutic value in patients with glaucoma due to the potential underlying vascular dysregulation pathogenesis. Researchers have shown promising results with Mg in the treatment of patients with glaucoma. Gaspar et al. [83] evaluated the effect of oral Mg therapy in 10 patients with glaucoma and 121.5 mg Mg was given twice daily for one month. Video-nailfold-capillaroscopy was used to evaluate peripheral capillary blood flow. At the end of 4 weeks of treatment, both visual field and peripheral blood flow were improved. They concluded that Mg supplementation seemed to have a beneficial effect on the visual field in patients with glaucoma. In another study, Aydin et al. [84] investigated the efficacy of oral Mg therapy on visual field perimetry indices and ocular blood flow in only NTG patients in a prospective controlled randomized clinical trial. Fifteen NTG patients received 300 mg oral Mg for one month and blood flow velocity of orbital vessels such as ophthalmic, posterior ciliary, and central retinal arteries was measured by color Doppler imaging. After one month, the improvements in visual field mean deviation and pattern standard deviation were found statistically significant in the study group compared to control group. They did not report any significant change in ocular blood flow parameters measured by color Doppler imaging. The authors speculated that mechanisms other than increased ocular blood flow may be responsible for the improvement in the visual field. Regulation of perfusion abnormality caused by vascular dysregulation may be the likely mechanism that Mg improved the ocular blood flow. Although only two clinical studies supported the beneficial role of Mg in the treatment of glaucoma, further studies with larger sample size and long term results would be valuable to better understand the impact of Mg in the management of glaucoma.

## 7. Conclusion

In conclusion, Mg is of critical importance in regulating cellular functions of the ocular tissues. The association of Mg levels with the pathogenesis of glaucoma may be attributed to Mg serving highly important roles as a cofactor for several enzymes involving membrane-associated ATPases, modulator of vessel smooth muscle contraction, and regulator in oxidative stress pathways. Therefore, Mg may have a great potential to come into clinical use in the management of glaucoma. The review of current literature supports the need of further investigations to evaluate the role of Mg as a supportive approach in glaucoma.

## References

[1] Weinreb R. N., Khaw P. T. (2004). Primary open-angle glaucoma. *The Lancet*.

[2] Mozaffarieh M., Flammer J. (2013). New insights in the pathogenesis and treatment of normal tension glaucoma. *Current Opinion in Pharmacology*.

[3] Mozaffarieh M., Flammer J. (2007). Is there more to glaucoma treatment than lowering IOP?. *Survey of Ophthalmology*.

[4] Lichter P. R., Musch D. C., Gillespie B. W. (2001). Interim clinical outcomes in the collaborative initial glaucoma treatment study comparing initial treatment randomized to medications or surgery. *Ophthalmology*.

[5] Anderson D. R., Drance S. M., Schulzer M. (2003). Factors that predict the benefit of lowering intraocular pressure in normal tension glaucoma. *American Journal of Ophthalmology*.

[6] Kaiser H. J., Schoetzau A., Stumpfig D., Flammer J. (1997). Blood-flow velocities of the extraocular vessels in patients with high- tension and normal-tension primary open-angle glaucoma. *American Journal of Ophthalmology*.

[7] Grunwald J. E., Piltz J., Hariprasad S. M., DuPont J. (1998). Optic nerve and choroidal circulation in glaucoma. *Investigative Ophthalmology and Visual Science*.

[8] Flammer J., Drance S. M. (1983). Reversibility of a glaucomatous visual field defect after acetazolamide therapy. *Canadian Journal of Ophthalmology*.

[9] Cellini M., Possati G. L., Caramazza N., Profazio V., Caramazza R. (1997). The use of flunarizine in the management of low-tension glaucoma: a color doppler study. *Acta Ophthalmologica Scandinavica*.

[10] Boehm A. G., Breidenbach K. A., Pillunat L. E., Bernd A. S., Mueller M. F., Koeller A. U. (2003). Visual function and perfusion of the optic nerve head after application of centrally acting calcium-channel blockers. *Graefe's Archive for Clinical and Experimental Ophthalmology*.

[11] Shahsuvaryan M. L. (2013). Glaucomatous optic neuropathy management: the role of neuroprotective agents. *Medical Hypothesis, Discovery & Innovation Ophthalmology*.

[12] Mayama C. (2013). Calcium channels and their blockers in intraocular pressure and glaucoma. *European Journal of Pharmacology*.

[13] Kaiser H. J., Stümpfig D., Flammer J. (1995). Short- Term effect of dipyridamole on blood flow velocities in the extraocular vessels. *International Ophthalmology*.

[14] Neufeld A. H., Sawada A., Becker B. (1999). Inhibition of nitric-oxide synthase 2 by aminoguanidine provides neuroprotection of retinal ganglion cells in a rat model of chronic glaucoma. *Proceedings of the National Academy of Sciences of the United States of America*.

[15] Quaranta L., Bettelli S., Uva M. G., Semeraro F., Turano R., Gandolfo E. (2003). Effect of Ginkgo biloba extract on preexisting visual field damage in normal tension glaucoma. *Ophthalmology*.

[16] Eckert A., Keil U., Kressmann S. (2003). Effects of EGb 761 Ginkgo biloba extract on mitochondrial function and oxidative stress. *Pharmacopsychiatry*.

[17] Saris N.-E. L., Mervaala E., Karppanen H., Khawaja J. A., Lewenstam A. (2000). Magnesium: an update on physiological, clinical and analytical aspects. *Clinica Chimica Acta*.

[18] Iseri L. T., French J. H. (1984). Magnesium: nature's physiologic calcium blocker. *American Heart Journal*.

[19] Agarwal R., Iezhitsa L., Agarwal P. (2014). Pathogenetic role of magnesium deficiency in ophthalmic diseases. *Biometals*.

[20] Elin R. J. (1994). Magnesium: the fifth but forgotten electrolyte. *American Journal of Clinical Pathology*.

[21] Barbagallo M., Dominguez L. J. (2010). Magnesium and aging. *Current Pharmaceutical Design*.

[22] Kupetsky-Rincon E. A., Uitto J. (2012). Magnesium: novel applications in cardiovascular disease—a review of the literature. *Annals of Nutrition and Metabolism*.

[23] Huerta M. G., Roemmich J. N., Kington M. L. (2005). Magnesium deficiency is associated with insulin resistance in obese children. *Diabetes Care*.

[24] Chang J. J., Mack W. J., Saver J. L., Sanossian N. (2014). Magnesium: potential roles in neurovascular disease. *Frontiers in Neurology*.

[25] Musso C. G. (2009). Magnesium metabolism in health and disease. *International Urology and Nephrology*.

[26] Smith J. M., Lowe R. F., Fullerton J., Currie S. M., Harris L., Felker-Kantor E. (2013). An integrative review of the side effects related to the use of magnesium sulfate for pre-eclampsia and eclampsia management. *BMC Pregnancy and Childbirth*.

[27] Gong H., Amemiya T., Takaya K. (2001). Retinal changes in magnesium-deficient rats. *Experimental Eye Research*.

[28] Katsanos K. H., Elisaf M., Bairaktari E., Tsianos E. V. (2001). Severe hypomagnesemia and hypoparathyroidism in Kearns-Sayre syndrome. *The American Journal of Nephrology*.

[29] Liang S. Y., Lee L. R. (2010). Retinitis pigmentosa associated with hypomagnesaemia. *Clinical and Experimental Ophthalmology*.

[30] Gong H., Takami Y., Amemiya T. (2003). Ultrastructure of the optic nerve in magnesium-deficient rats. *Ophthalmic Research*.

[31] Kirkpatrick H. (1920). The use of magnesium sulphate as a local application in inflammation of the conjunctiva and cornea. *British Journal of Ophthalmology*.

[32] Gong H., Takami Y., Kitaoka T., Amemiya T. (2003). Corneal changes in magnesium-deficient rats. *Cornea*.

[33] Thalasselis A., Taie H. F., Etchepareborda J., Selim A. (1988). Keratoconus, magnesium deficiency, type A behavior, and allergy. *The American Journal of Optometry and Physiological Optics*.

[34] Agarwal R., Iezhitsa I. N., Agarwal P., Spasov A. A. (2013). Mechanisms of cataractogenesis in the presence of magnesium deficiency. *Magnesium Research*.

[35] Hatwal A., Gujral A. S., Bhatia R. P. S., Agrawal J. K., Bajpai H. S. (1989). Association of hypomagnesemia with diabetic retinopathy. *Acta Ophthalmologica*.

[36] Grieshaber M. C., Mozaffarieh M., Flammer J. (2007). What is the link between vascular dysregulation and glaucoma?. *Survey of Ophthalmology*.

[37] Flammer J., Orgül S. (1998). Optic nerve blood-flow abnormalities in glaucoma. *Progress in Retinal and Eye Research*.

[38] Kaiser H. J., Flammer J. (1991). Systemic hypotension: a risk factor for glaucomatous damage?. *Ophthalmologica*.

[39] Kaiser H. J., Flammer J., Graf T., Stumpfig D. (1993). Systemic blood pressure in glaucoma patients. *Graefe's Archive for Clinical and Experimental Ophthalmology*.

[40] Nakabayashi M. (2004). Review of the ischemia hypothesis for ocular hypertension other than congenital glaucoma and closed-angle glaucoma. *Ophthalmologica*.

[41] Flammer J., Konieczka K., Flammer A. J. (2013). The primary vascular dysregulation syndrome: implications for eye diseases. *The EPMA Journal*.

[42] Flammer J., Haefliger I. O., Orgül S., Resink T. (1999). Vascular dysregulation: a principal risk factor for glaucomatous damage?. *Journal of Glaucoma*.

[43] Wierzbowska J., Wierzbowski R., Stankiewicz A., Siesky B., Harris A. (2012). Cardiac autonomic dysfunction in patients with normal tension glaucoma: 24-h heart rate and blood pressure variability analysis. *British Journal of Ophthalmology*.

[44] Gugleta K., Orgül S., Hasler P. W., Picornell T., Gherghel D., Flammer J. (2003). Choroidal vascular reaction to hand-grip stress in subjects with vasospasm and its relevance in glaucoma. *Investigative Ophthalmology and Visual Science*.

[45] Landsberg L. (1999). Role of the sympathetic adrenal system in the pathogenesis of the insulin resistance syndrome. *Annals of the New York Academy of Sciences*.

[46] Stewart R. M. K., Clearkin L. G. (2008). Insulin resistance and autoregulatory dysfunction in glaucoma and retinal vein occlusion. *The American Journal of Ophthalmology*.

[47] Oh S. W., Lee S., Park C., Kim D. J. (2005). Elevated intraocular pressure is associated with insulin resistance and metabolic syndrome. *Diabetes/Metabolism Research and Reviews*.

[48] Sjogren A., Floren C.-H., Nilsson A. (1986). Magnesium deficiency in IDDM related to level of glycosylated hemoglobin. *Diabetes*.

[49] Le A., Mukesh B. N., McCarty C. A., Taylor H. R. (2003). Risk factors associated with the incidence of open-angle glaucoma: the visual impairment project. *Investigative Ophthalmology and Visual Science*.

[50] de Voogd S., Ikram M. K., Wolfs R. C. W. (2006). Is diabetes mellitus a risk factor for open-angle glaucoma?. The Rotterdam study. *Ophthalmology*.

[51] Tielsch J. M., Katz J., Quigley H. A., Javitt J. C., Sommer A. (1995). Diabetes, intraocular pressure, and primary open-angle glaucoma in the Baltimore Eye Survey. *Ophthalmology*.

[52] Lee N. Y., Park H. L., Park C. K., Ahn M. D. (2012). Analysis of systemic endothelin-1, matrix metalloproteinase-9, macrophage chemoattractant protein-1, and high-sensitivity C-reactive protein in normal-tension glaucoma. *Current Eye Research*.

[53] Leibovitch I., Kurtz S., Kesler A., Feithliher N., Shemesh G., Sela B. (2005). C-reactive protein levels in normal tension glaucoma. *Journal of Glaucoma*.

[54] Gathwala G. (2001). Neuronal protection with pagnesium. *Indian Journal of Pediatrics*.

[55] Tezel G. (2006). Oxidative stress in glaucomatous neurodegeneration: Mechanisms and consequences. *Progress in Retinal and Eye Research*.

[56] Neufeld A. H., Hemandez R., Gonzalez M. (1997). Nitric oxide synthase in the human glaucomatous optic nerve head. *Archives of Ophthalmology*.

[57] Retamal M. A., Yin S., Altenberg G. A., Reuss L. (2009). Modulation of Cx46 hemichannels by nitric oxide. *American Journal of Physiology—Cell Physiology*.

[58] Shoshani Y. Z., Harris A., Shoja M. M. (2012). Endothelin and its suspected role in the pathogenesis and possible treatment of glaucoma. *Current Eye Research*.

[59] Noske W., Hensen J., Wiederholt M. (1997). Endothelin-like immunoreactivity in aqueous humor of patients with primary open-angle glaucoma and cataract. *Graefe's Archive for Clinical and Experimental Ophthalmology*.

[60] Prasanna G., Krishnamoorthy R., Clark A. F., Wordinger R. J., Yorio T. (2002). Human optic nerve head astrocytes as a target for endothelin-1. *Investigative Ophthalmology and Visual Science*.

[61] Dettmann E. S., Lüscher T. F., Flammer J., Haefliger I. O. (1998). Modulation of endothelin-1-induced contractions by magnesium/calcium in porcine ciliary arteries. *Graefe’s Archive for Clinical and Experimental Ophthalmology*.

[62] Orgul S., Cioffi G. A., Wilson D. J., Bacon D. R., Van Buskirk E. M. (1996). An endothelin-1 induced model of optic nerve ischemia in the rabbit. *Investigative Ophthalmology & Visual Science*.

[63] Toriu N., Sasaoka M., Shimazawa M., Sugiyama T., Hara H. (2001). Effects of lomerizine, a novel Ca^2+^ channel blocker, on the normal and endothelin-1-disturbed circulation in the optic nerve head of rabbits. *Journal of Ocular Pharmacology and Therapeutics*.

[64] DeWitt C. R., Waksman J. C. (2004). Pharmacology, pathophysiology and management of calcium channel blocker and *β*-blocker toxicity. *Toxicological Reviews*.

[65] Shechter M., Sharir M., Paul M. J. (2000). Oral magnesium therapy improves endothelial function in patients with coronary artery disease. *Circulation*.

[66] Touyz R. M. (2003). Role of magnesium in the pathogenesis of hypertension. *Molecular Aspects of Medicine*.

[67] Laurant P., Berthelot A. (1996). Endothelin-1-induced contraction in isolated aortae from normotensive and DOCA-salt hypertensive rats: effect of magnesium. *British Journal of Pharmacology*.

[68] Ishiguro S., Matsuyama T., Sakaguchi H., Nishio A. (1997). Ex vivo study of the increased sensitivity to NO of endothelium-denuded thoracic aortas isolated from dietary magnesium-deficient rats. *Magnesium Research*.

[69] Barbagallo M., Dominguez L. J., Galioto A., Pineo A., Belvedere M. (2010). Oral magnesium supplementation improves vascular function in elderly diabetic patients. *Magnesium Research*.

[70] Yang Z. W., Gebrewold A., Nowakowski M., Altura B. T., Altura B. M. (2000). Mg^2+^-induced endothelium-dependent relaxation of blood vessels and blood pressure lowering: role of NO. *American Journal of Physiology: Regulatory Integrative and Comparative Physiology*.

[71] Shareef S., Sawada A., Neufeld A. H. (1999). Isoforms of nitric oxide synthase in the optic nerves of rat eyes with chronic moderately elevated intraocular pressure. *Investigative Ophthalmology and Visual Science*.

[72] Thoreson W. B., Witkovsky P. (1999). Glutamate receptors and circuits in the vertebrate retina. *Progress in Retinal and Eye Research*.

[73] Vorwerk C. K., Lipton S. A., Zurakowski D., Hyman B. T., Sabel B. A., Dreyer E. B. (1996). Chronic low-dose glutamate is toxic to retinal ganglion cells: toxicity blocked by memantine. *Investigative Ophthalmology and Visual Science*.

[74] Chrysostomou V., Rezania F., Trounce I. A., Crowston J. G. (2013). Oxidative stress and mitochondrial dysfunction in glaucoma. *Current Opinion in Pharmacology*.

[75] Lee D., Shim M. S., Kim K. Y. (2014). Coenzyme Q10 inhibits glutamate excitotoxicity and oxidative stress-mediated mitochondrial alteration in a mouse model of glaucoma. *Investigative Ophthalmology & Visual Science*.

[76] Novelli A., Reilly J. A., Lysko P. G., Henneberry R. C. (1988). Glutamate becomes neurotoxic via the N-methyl-D-aspartate receptor when intracellular energy levels are reduced. *Brain Research*.

[77] Sucher N. J., Lipton S. A., Dreyer E. B. (1997). Molecular basis of glutamate toxicity in retinal ganglion cells. *Vision Research*.

[78] Sucher N. J., Lei S. Z., Lipton S. A. (1991). Calcium channel antagonists attenuate NMDA receptor-mediated neurotoxicity of retinal ganglion cells in culture. *Brain Research*.

[79] Mills B. J., Lindeman R. D., Lang C. A. (1986). Magnesium deficiency inhibits biosynthesis of blood glutathione and tumor growth in the rat. *Proceedings of the Society for Experimental Biology and Medicine*.

[80] Szabo M. E., Droy-Lefaix M. T., Doly M., Braquet P. (1992). Ischaemia- and reperfusion-induced Na+, K+, Ca2+ and Mg2+ shifts in rat retina: effects of two free radical scavengers, SOD and EGB 761. *Experimental Eye Research*.

[81] Alberty R. A. (1968). Effect of pH and metal ion concentration on the equilibrium hydrolysis of adenosine triphosphate to adenosine diphosphate.. *Journal of Biological Chemistry*.

[82] George G. A., Heaton F. W. (1975). Changes in cellular composition during magnesium deficiency. *Biochemical Journal*.

[83] Gaspar A. Z., Gasser P., Flammer J. (1995). The influence of magnesium on visual field and peripheral vasospasm in glaucoma. *Ophthalmologica*.

[84] Aydin B., Önol M., Hondur A. (2010). The effect of oral magnesium therapy on visual field and ocular blood flow in normotensive glaucoma. *European Journal of Ophthalmology*.

